# ID 287 - O que Podemos Esperar para o Futuro do Tratamento da Hemoglobinúria Paroxística Noturna (HPN) no Sistema Único de Saúde (SUS)?

**DOI:** 10.5327/2237-9622.2025.v34s1.287

**Published:** 2025-11-25

**Authors:** Cecília de Oliveira Carvalho Faria, Cecília de Oliveira Carvalho Faria, Alícia Dorneles Dornelles, Bruna Bento dos Santos, Mônica Vinhas de Souza, Aldenora Ximenes, Júlia Cordeiro Milke, Ida Vanessa Doederlein Schwartz

**Keywords:** doenças raras, hemoglobinuria paroxística noturna, economia e organizações de saúde

## Abstract

**Introdução:**

A hemoglobinúria paroxística noturna (HPN) é um distúrbio clonal raro de células-tronco hematopoiéticas que se manifesta com anemia hemolítica, trombose e insuficiência medular. A deficiência dos reguladores do complemento na membrana das células hematopoiéticas torna essas células suscetíveis a danos, com alta morbidade e mortalidade. A introdução dos inibidores C5 revolucionou o tratamento, melhorando significativamente a qualidade de vida desses pacientes. O tratamento no SUS inclui eculizumabe e transfusões sanguíneas. No entanto, desafios permanecem, como hemólise residual, levando à busca por novas abordagens terapêuticas. Assim sendo, este estudo teve como objetivo identificar e descrever tecnologias avaliadas pela Comissão Nacional de Incorporação de Tecnologias no SUS (Conitec) para o tratamento da HPN e mapear tecnologias emergentes para o tratamento da doença.

**Método:**

Trata-se de um estudo descritivo, no qual foram realizadas buscas manuais na página eletrônica da Conitec para identificar tecnologias de tratamento da HPN demandadas à Comissão. Para mapear o horizonte tecnológico, foram realizadas buscas estruturadas no ClinicalTrials.gov e Cortellis™. A pesquisa foi conduzida em 23 de maio de 2024. Foram considerados estudos clínicos de fases 2, 3 ou 4 no ClinicalTrials.gov, que testaram ou estão testando medicamentos para HPN. Além disso, foram incluídas tecnologias registradas nos últimos cinco anos para HPN na Agência Nacional de Vigilância Sanitária (Anvisa), (EMA) ou (FDA), que não estão disponíveis no SUS.

**Resultados:**

Foram identificadas cinco tecnologias demandadas à Conitec: a incorporação da vacina adsorvida Meningocócica B (recombinante) para pacientes com HPN em uso de eculizumabe (não incorporada); a exclusão do eculizumabe para o tratamento da HPN (não excluído); a incorporação do ravulizumabe para pacientes maiores de 14 anos, sejam virgens de tratamento ou em uso prévio de eculizumabe (incorporado); a pegcetacoplana para o tratamento de adultos com HPN previamente tratados com inibidores do complemento (em análise); e a pegcetacoplana para o tratamento de adultos com HPN sem tratamento prévio com inibidores do complemento (em análise). No horizonte tecnológico, foram identificadas 13 tecnologias. Uma delas, a pegcetacoplana, como mencionado, já está em análise pela Conitec. O iptacopan e o danicopan receberam aprovação pelo FDA e pela EMA, enquanto o crovalimabe está em fase de pré-registro nas duas agências. As demais estão em fases 2/3 de desenvolvimento, algumas já com designação de droga-órfã.

**Conclusão:**

As análises realizadas revelam um cenário dinâmico, com muitos medicamentos surgindo para tratar a HPN. É esperado que a Conitec continue a receber propostas de incorporação de novas terapias para essa condição. A monitorização ativa associada à avaliação das tecnologias que despontam é essencial para o manejo otimizado dessa doença complexa, potencializando o uso mais eficiente dos recursos finitos do SUS.

